# Comparison of different adjuvant analgesia for paravertebral block in video-assisted thoracoscopic surgery: A double-blind randomized controlled trial

**DOI:** 10.1371/journal.pone.0322589

**Published:** 2025-05-02

**Authors:** Rong Tang, Wen-sheng Lu, Hai-lian Zhong, Fang Wu, Yu-qian Liu

**Affiliations:** Department of Anesthesiology, The Second Hospital of Lanzhou University, Lanzhou, China; Stanford University School of Medicine, UNITED STATES OF AMERICA

## Abstract

**Background:**

Local anaesthetic adjuvants have been shown to provide better pain relief and extend the duration of analgesia. But little information is available on which adjuvants are more effective in block extension for thoracic paravertebral block (TPVB) during video-assisted thoracoscopic surgery (VATS). This study aimed to compare the analgesic efficacies of different adjuvants with 0.375% ropivacaine in ultrasound-guided TPVB for VATS.

**Methods:**

A total of 120 patients who underwent VATS at the study hospital in 2022–2023 were recruited and randomly divided into four groups, including the control group (Group R), which received 20 ml ropivacaine 0.375% for TPVB, and the intervention groups: Group D (0.375% ropivacaine with 1 µg/kg dexmedetomidine in a total of 20 ml for TPVB), group E (0.375% ropivacaine with 0.1 mg/kg dexamethasone in a total of 20 ml for TPVB), and group S (0.375% ropivacaine with 0.5ug/ml sufentanil in a total of 20 ml for TPVB). They received a single-injection ultrasound-guided unilateral T5-level TPVB. The primary clinical outcomes were visual analogue scale (VAS) pain scores at rest and during coughing at 4, 8, 12, 16, 20, 24, and 48 h postoperatively. Secondary outcomes included opioid consumption, number of patient-controlled intravenous analgesics (PCIA) used within 48 h, postoperative rescue analgesia, side effects, quality of recovery after surgery, and length of hospital stay.

**Results:**

We found that the VAS pain scores in the resting state during the postoperative period at 12–20 h were lower in Group D than in Group R (*P* < 0.05). The resting VAS pain scores of Groups E and S were lower than those of Group R during the postoperative period of 12–16 h (*P* < 0.05). Similar differences were observed in the pain scores during coughing at the same time points. Additionally, the co-administration of adjuvants with ropivacaine were associated with decreased consumption of postoperative opioids, less frequent PCIA use, and earlier time to ambulation. The intergroup pairwise comparisons showed that dexmedetomidine was more effective than dexamethasone and sufentanil in reducing additional postoperative analgesics, the length of chest tube placement and length of hospital stay. Subsequently, we administered questionnaires on the quality of recovery, which was positively correlated with the postoperative analgesic effect.

**Conclusions:**

These findings suggest that adjuvant analgesia for TPVB can improve postoperative pain in patients undergoing VATS. However, compared with dexamethasone and sufentanil, dexmedetomidine is more effective in extending the duration of analgesia, reducing the demand of postoperative analgesics, improving the quality of recovery at 48 h postoperatively and shorting the length of hospital stay.

## Introduction

Moderate to severe postoperative pain in video-assisted thoracoscopic surgery (VATS) patients frequently results in complications such as atelectasis and hypostatic pneumonia. Effective post-operative analgesia reduces the incidence of early post-operative complications. Epidural block analgesia is the gold standard for pain management after thoracic surgery. However, serious complications, including dural puncture, epidural hematoma, and hypotension may arise [1,2].

With the development of the regional nerve block technique, thoracic paravertebral block (TPVB) is increasingly used in patients undergoing VATS [3]. Several studies have demonstrated that patients who undergo TPVB have a significantly lower postoperative opioid demand and higher postoperative satisfaction than those who do not undergo TPVB [4,5]. However, the duration of postoperative analgesia can only be maintained for 6–8 h even when medium and long-acting local anaesthetics are used [6,7]. The potential to prolong the duration of analgesia after a single TPVB injection is especially important.

To provide a longer duration of analgesia, many adjuvants have been used in combination with local anaesthetics for peripheral and neuroaxial blocks [8–12]. Dexmedetomidine is a highly selective short-acting alpha-2 agonist with centrally mediated sedative, anti-anxiety, perioperative sympathetic excitation and hypnotic inhibition effects [13]. In recent years, some studies have shown that the addition of dexmedetomidine to local anaesthetics in peripheral nerve blocks can extend the duration of the block and reduce the need for analgesia [14,15]. Dexamethasone, a long-acting glucocorticoid, is one of the most studied and widely used local anaesthesia adjuvants [16]. One study found that the addition of 8 mg dexamethasone to a local anaesthetic for an interscalene block almost doubled the time to the first need for pain medication [17]. In addition, dexamethasone-assisted peripheral nerve block is effective in most patients [18,19]. Opioids are a type of compound that exerts analgesic effects by binding to opioid receptors. Several studies have combined highly lipophilic opioids, such as fentanyl and sufentanil, with local anaesthetics to epidural for enhanced analgesic efficacy [20,21]. A prospective randomised trial in breast surgery showed that the analgesic effect of TPVB using fentanyl combined with levobupivacaine was superior to that of levobupivacaine alone [22]. Sufentanil demonstrates 5–10-fold greater analgesic potency compared to fentanyl, with its high lipophilicity facilitating rapid diffusion across neural sheaths for enhanced binding affinity to μ-opioid receptors, thereby potentiating the neural-targeting efficacy of local anesthetics. This pharmacodynamic synergy supports its use in nerve block protocols, where rapid receptor engagement and prolonged analgesic effects optimize perioperative pain management‌. However, little information is available on which adjuvants are more effective in block extension for TPVB in VATS.

The purpose of this study was to investigate the analgesic efficacies of different adjuvants with ropivacaine in ultrasound-guided TPVB for VATS. The researchers hypothesized that ropivacaine with adjuvants in TPVB would be able to decrease the postoperative VAS score, reduce the consumption of opioids and improve the quality of recovery 48h after VATS. Furthermore, compared with dexamethasone and sufentanil, dexmedetomidine may be more effective in extending the duration of analgesia and reducing the length of hospital stay.

## Methods

This study was approved by the Ethics Committee of Lanzhou University Second Hospital, Lanzhou, Gansu Province on 01/09/2021 (2021A-525) and registered in the Chinese Clinical Trial Registry (ChiCTR2000038037). All enrolled patients signed a written informed consent form prior to the surgery. A total of 135 patients aged 18–70 years old with American Society of Anesthesiologists (ASA) I–III who were scheduled for VATS under general anaesthesia between January 15th, 2022 to December 10th, 2023 were enrolled in this study.

Patients excluded from the study included those with puncture site infection, coagulopathy disorders, uncontrolled diabetes mellitus, ropivacaine or other amide local anaesthetic drug allergy or hypersensitivity, severe heart and lung disease, arrhythmia, liver and kidney insufficiency or mental illness, history of chronic pain or long-term history of taking analgesics, and known allergies to investigational drugs. The withdrawal criteria included severe complications or accidents during the perioperative period or anaesthesia and those who did not cooperate well with the visual analogue scale (VAS) scores.

In this randomized controlled study, we employed a completely randomized balanced design. The SAS v9.4 statistical software was utilized to generate a random number table, and the 120 participants were randomly allocated into four equal groups. We did not actively control external variables, instead relying solely on random probability to achieve an approximate balance of baseline characteristics between the groups. The distribution results were sealed in an opaque envelope and opened immediately prior to the peripheral nerve block, which was performed by a physician not directly involved in patient care. On the day of the surgery, the main researcher handed the envelope to the assistant anaesthesiologist to prepare the test liquid. The contents of the solution were confidential to the patient and anaesthesiologist at all times.

Thirty minutes before surgery, patients were admitted to the pre-anaesthesia room, and pulse oxygen saturation, non-invasive arterial blood pressure (BP), and electrocardiograms were routinely monitored with supplemental oxygen delivery via a nasal cannula. Subsequently, 3 mg of midazolam was administered intravenously for sedation, and the patients were placed in the lateral decubitus position. The TPVB was performed in the pre-anaesthesia room by an experienced regional anaesthesiologist. A high-frequency linear ultrasound transducer (GE LOGIQe, Wauwatosa, WI, USA) was placed along the sagittal axis of the horizontal midline of the T5 vertebral body. The spinous processes were first observed, and the probe was then moved laterally to observe the transverse process and superior costotransverse ligament approximately 3 cm from the midline. A 10 cm 22-G ultrasound visual needle (Contiplex; B Braun, Melsungen, Germany) was guided into the paravertebral space using an in-plane technique, and the correct position of the needle was confirmed by injecting 2–3 ml of saline solution, after which the anaesthetic solution was administered. Heart rate and BP were measured after injection. Prompt treatment was required if bradycardia or hypotension occurred.

After entering the operating room, all patients underwent standard electrocardiography, partial oxygen pulse oximetry, invasive BP measurements, and bispectral index (BIS) monitoring. The induction of anaesthesia was performed with intravenous propofol 1.5–2.0 mg/kg, sufentanil 0.5ug/kg, and cisatracurium 0.2 mg/kg. After induction, double-lumen endotracheal intubation was performed and mechanical ventilation initiated. The BIS value was maintained between 40 and 60 by continuous infusion of propofol 4–6 mg/kg/h and remifentanil 0.15–0.3µg/kg/min. When necessary, 0.1 mg/kg cisatracurium was administered for muscle relaxation during the operation. The doses were adjusted based on the patient’s condition, BIS, and course of the surgery. The haemodynamics during surgery were maintained within 30% of baseline levels.

All patients received 3-port single-intercostal VATS; the thoracoscopic port was performed at the 6–7th intercostal of the midaxillary line, the main operating port at the 4–5th intercostal of the anterior axillary line, and the secondary operating port at the 7th intercostal of the posterior axillary line; and the same postoperative care was provided by the surgical team. The same surgical team performed VATS on all patients in the lateral position. A 24 Fr chest tube was inserted through the thoracoscopic port and connected to an underwater-sealed bottle.

After surgery, patients were transferred to the post anaesthesia care unit (PACU) with PCIA which consisted of hydrogen morphine ketone 0.1 mg/ml, butorphanol 50 µg/ml, and ondansetron 16 mg. The PCIA pump was programmed to deliver a bolus amount of 2 mL and a background dose of 2.5 mL/h, and had a lockout period of 15 min. Pain severity was measured every 10 min in the PACU using the VAS from zero (no pain) to 10 (maximum pain) and again at 4, 8, 12, 16, 20, 24, and 48 h after surgery in the surgical ward. If the VAS score at rest exceeded 4, a bolus injection of 0.2 mg hydrogen morphine ketone and 100 µg butorphanol was given through the PCIA to alleviate the pain with a 15-minute lockout period. If the pain didn’t relieve after PCIA, patients received ketorolac tromethamine 60 mg as a rescue analgesic, which should be halved to 30mg for patients over 65 years old or weighing less than 50 kg. Re-evaluation was performed after 30 minutes of administration, if the VAS score was still greater than 4. Supplemental oral oxycodone 5mg was provided when necessary and practical. The oxycodone consumption was included in the post-operative opioid use and was eventually converted to morphine milligram equivalents (MME).

Postoperative care and evaluation were conducted by a researcher who was blinded to the study group. The primary outcomes were 4, 8, 12, 16, 20, 24 and 48 h pain intensity at rest and during coughing after surgery. Secondary outcomes included opioid consumption, use of rescue analgesics within 48 h postoperatively, PCIA press times within 48 h postoperatively, time to first ambulation, indwelling chest tube time, length of hospital stay, and incidence of postoperative side effects. The time to first ambulation was defined as the time from surgery until the patient independently got out of bed. In addition, patients were questioned at 24 h, 48 h, 72 h, and 1 week postoperatively using the 15-item Patient-Related Quality of Recovery Questionnaire (QoR-15) to evaluate the quality of recovery which was correlated with the postoperative analgesic effect.

### Statistical analysis

The sample size for this study was determined based on preliminary data collected from 20 patients randomly assigned to 5 subjects in each group. The results showed that the resting pain at 12 h postoperatively was 3.01 (0.51) in group R and 2.36 (0.53), 2.48 (0.55), and 2.62 (0.52) in groups D, E and S, respectively. We estimated the sample size based on the mean (standard deviation [SD]) with the least difference from the control group. We calculated the maximum sample size of 26 patients in each group for a type I error of 0.05 and a type II error of 0.1, translating to a 90% power to detect this difference. Ultimately, we recruited 30 patients from each group, for a total of 120 patients, considering possible dropouts and incomplete follow-up.

Statistical analyses were performed using SPSS software (version 25.0; IBM, USA).

Assumptions of parametric tests (normality, homogeneity of variance) were verified using Shapiro-Wilk and Levene’s test‌. The measurement data were expressed as mean ± standard deviation (SD) or median [inter-quartile range (IQR)]. The count data were expressed as frequencies or percentages. A multigroup comparison of the normally distributed measurement data with homogeneity of variance was conducted using one-way analysis of variance (ANOVA), and the Tukey-Kramer method was used to compare the intergroup pairwise. The Kruskal-Wallis H-test was employed to compare measurement data that did not adhere to a normal distribution or exhibited heterogeneous variance among multiple groups. And the Dunn’s test was utilized for intergroup pairwise comparisons. The original p-value was adjusted using the Bonferroni method, thereby more rigorously controlling the type I error rate. The Chi-squared (χ^2^) test was used to compare categorical and count data, and P < 0.05 was considered statistically significant.

## Results

**Fig 1:**
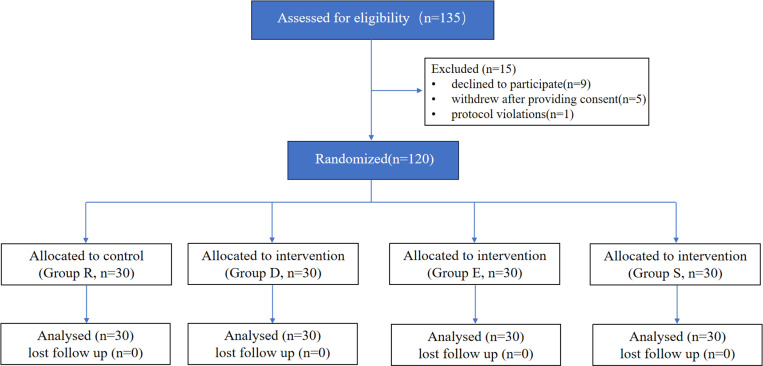
Flow diagram of study. Group R, (ropivacaine), Group D (ropivacaine with dexmedetomidine), Group E (ropivacaine with dexamethasone), Group S (ropivacaine with sufentanil).

Among the 135 patients evaluated for eligibility, nine declined to participate, five withdrew after providing consent, and one was excluded due to protocol deviation, mainly because the patient did not take the analgesic prescribed in the trial protocol. A total of 120 patients were included in the analysis and completed the postoperative follow-up (Fig 1).

There were no significant differences in sex, age, ASA physical status, body mass index, or surgical characteristics among the four groups. (*P *> 0.05) (Table 1). However, there were significant differences in the chest tube indwelling time (*P* = 0.01) and the length of hospital stay (*P* = 0.04) between the four groups. A more detailed pairwise comparison among the groups revealed that the duration of thoracic tube retention (*P* = 0.0072) and the overall hospital stay (*P* = 0.0341) were notably shorter in Group D when compared to Group R. And no substantial differences were observed across the remaining groups (*P* > 0.05).

**Table 1:** Comparison of baseline characteristics and surgery duration between the four groups.

Category	Groups				*χ2/F/H*	*P*
	Group R (n = 30)	Group D (n = 30)	Group E (n = 30)	Group S (n = 30)		
Age (years)	58.40 ± 6.89	56.23 ± 8.76	55.47 ± 8.63	57.35 ± 7.50	1.17	0.32
Weight (Kg)	65.67 ± 7.72	65.76 ± 7.85	65.03 ± 8.09	66.82 ± 8.12	0.05	0.98
Gender (male/female)	15/15	16/14	15/15	14/16	0.27	0.96
Height (cm)	166.53 ± 7.87	166.73 ± 9.23	166.20 ± 8.73	165.20 ± 9.63	0.15	0.93
ASA status					0.71	0.87
Ⅰ	12	11	13	10		
Ⅱ	18	19	17	20		
Anesthesia time(min)	165.83 ± 27.01	161.38 ± 31.55	164.33 ± 32.75	163.66 ± 32.95	0.32	0.81
Surgery time (min)	129.50 ± 23.94	130.50 ± 28.07	131.50 ± 25.07	128.50 ± 29.57	0.27	0.85
Chest tube indwelling time (h)	57.56 ± 4.56	53.43 ± 5.34^a^	54.83 ± 5.10	54.97 ± 4.31	11.32	0.01
Length of hospital stay (d)	7.47 ± 1.01	6.65 ± 1.24^ a^	6.83 ± 1.19	7.03 ± 1.16	8.34	0.04

Group R: ropivacaine, Group D: ropivacaine with dexmedetomidine, Group E: ropivacaine with dexamethasone, Group S: ropivacaine with sufentanil. Values are n, n (%), mean (standard deviation), unless otherwise noted. ASA, American Society of Anesthesiologists.

^a^refers to intergroup comparisons against Group R (adjusted *P* value <0.05).

**Fig 2:**
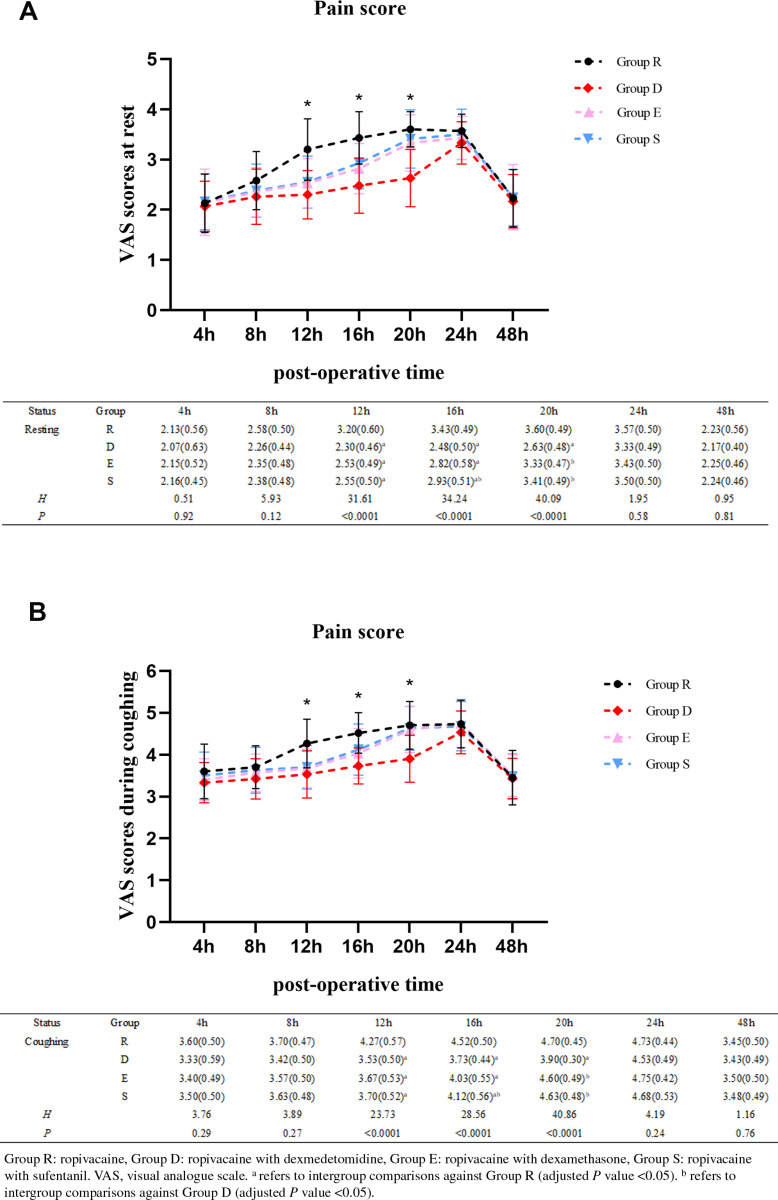
Comparison of VAS pain scores (A) at rest and (B) during coughing between the four groups. Data were expressed as mean (standard deviation).

All participants successfully underwent unilateral TPVB. No complications associated with regional anaesthesia were observed. The results of the Shapiro-Wilk test and Levene’s test indicated that the VAS scores displayed a non-normal distribution or exhibited heterogeneous variance among multiple groups at each designated time point following the operation. Therefore, the Kruskal-Wallis H-test was employed to compare measurement data and the results revealed that there were significant differences in the VAS scores at rest and during coughing among the four groups at 12h, 16h, and 20h postoperatively (*P* < 0.0001). Subsequently, the Dunn’s test was utilized for intergroup pairwise comparisons and demonstrated that in comparison with the Group R, the participants in Group D consistently exhibited significantly lower mean VAS scores during periods of rest at 12h, 16h, and 20h postoperatively (mean [SD], 2.3 [0.48] vs 3.2 [0.61], 2.48 [0.55] vs 3.43 [0.52], 2.63 [0.57] vs 3.60 [0.35], adjusted *P* value <0.0001). Similarly, the VAS scores at rest of patients in Group E were relatively lower than those of patients in Group R at 12 and 16 h postoperatively, (mean [SD], 2.53 [0.49] vs 3.2 [0.61], adjusted *P* value = 0.0008, 2.82 [0.50] vs 3.43 [0.52], adjusted *P* value = 0.0014). Concurrently, the pain score at rest in Group S was significantly lower than that in Group R at 12 and 16 h postoperatively (mean [SD], 2.55 [0.52] vs 3.2 [0.61], adjusted *P* value = 0.0019; 2.93 [0.51] vs 3.43 [0.52], adjusted *P* value = 0.0143). Moreover, compared with the same time points in Group D, the VAS scores were higher at 20 h postoperatively in Group E (mean [SD], 3.33 [0.56] vs 2.63 [0.57], adjusted *P* value = 0.0002), and the patients in Group S showed higher pain scores at both 16 and 20h postoperatively (mean [SD], 2.93 [0.51] vs 2.48 [0.55], adjusted *P* value = 0.0368; 3.41 [0.58] vs 2.63 [0.57], adjusted *P* value < 0.0001). However, there was no significant difference between Group E and S (*P* > 0.05). (Fig 2A). Similar differences were observed in the pain scores during coughing at the same time points (Fig 2B). In contrast, there were no significant differences in the VAS scores at 4, 8, 24, and 48 h postoperatively between the four groups.

The same method was used to compare secondary outcomes between the four groups. The results showed that when compared with Group R, the intervention groups showed lower opioid consumption MME at POD1 (mean [SD], 77.83 [3.69], 81.58 [3.69], 82.83 [6.94] vs 90.75 [7.93] mg, adjusted *P* value <0.0001) and POD2 (mean [SD], 75.83 [2.18], 78.75 [4.17], 79.83 [5.35] vs 83.91 [8.43] mg, adjusted *P* value <0.05). And there were also significant differences between Group D and Group S at POD1 (adjusted *P* value = 0.0141) and POD2 (adjusted *P* value = 0.0295). Moreover, in Group D, patients exhibited lower rescue consumption of ketorolac tromethamine within 48 h postoperatively compared with Group R (mean [SD], 45.00 [26.39] vs 65.00 [27.39] mg, adjusted *P* value = 0.0179). Additionally, the time to the first use of PCIA was significantly later in Groups D, E and S than that in Group R (mean [SD], 15.6 [3.6], 11.3 [4.7], 10.8 [4.3] vs 6.8 [2.7] h, adjusted *P* value <0.05), and the PCIA press times within 48 h postoperatively were also lower (median [IQR], 1(0–3), 2(0–4), 2(0–4) vs 4(2–5) times, adjusted *P* value <0.05). Furthermore, the time to first ambulation in Group D, E, and S were significantly shorter than those in Group R (adjusted *P* value <0.0001). Moreover, there were also significant differences between Group D and Group S (adjusted *P* value = 0.0032). (Table 2).

**Table 2:** Comparison of secondary outcomes between the groups.

Outcome	Groups				*χ* ^*2*^ */F/H*	*P*
	Group R (n = 30)	Group D (n = 30)	Group E (n = 30)	Group S (n = 30)		
Opioid consumption MME (mg)						
Intraoperative	171 ± 50.87	161 ± 36.15	164 ± 37.45	167 ± 40.7	0.31	0.81
PACU	7.88 ± 2.84	6.96 ± 2.11	7.37 ± 1.73	7.79 ± 1.91	1.12	0.34
POD 1 POD 2	90.75 ± 7.93 83.91 ± 8.43	77.83 ± 3.69^a^ 75.83 ± 2.18 ^a^	81.58 ± 5.89 ^a^ 78.75 ± 4.17 ^a^	82.83 ± 6.94 ^ab^ 79.83 ± 5.35 ^ab^	21.95 11.02	＜0.0001 ＜0.0001
Rescue analgesic within 48h ketorolac tromethamine(mg)	65.00 ± 27.39	45.00 ± 26.93^ a^	48.00 ± 25.26	55.00 ± 24.09	3.52	0.02
Time to first ambulation (h)	34.8 ± 6.5	21.6 ± 4.4 ^a^	24.6 ± 5.4 ^a^	26.8 ± 6.2 ^ab^	29.65	＜0.0001
Time to first use of PCIA(h)	6.8 ± 2.7	15.6 ± 3.6^ a^	11.3 ± 4.7^ ab^	10.8 ± 4.3^ ab^	25.56	＜0.0001
Times PCIA used within 48 h	4(2-5)	2(1–3) ^a^	3(2–4) ^ab^	3(2–4) ^ab^	41.83	＜0.0001

Group R: ropivacaine, Group D: ropivacaine with dexmedetomidine, Group E: ropivacaine with dexamethasone, Group S: ropivacaine with sufentanil. Values are presented as mean (standard deviation) apart from PCIA press times which is presented as median (inter-quartile range); MME, morphine milligram equivalents. POD, post-operative day. PACU, post anaesthesia care unit. PCIA, patient-controlled intravenous analgesia. VAS, visual analogue scale.

a refers to intergroup comparisons against Group R (adjusted *P* value <0.05).

b refers to intergroup comparisons against Group D (adjusted *P* value <0.05).

No respiratory depression, pulmonary infection, or neurotoxicity occurred in any of the groups. There were no significant differences in the incidence of hypotension, bradycardia, dizziness, or nausea and vomiting between the four groups within 48 h postoperatively (P > 0.05). (Table 3).

**Table 3:** Comparison of postoperative adverse events among the four groups.

Adverse events	Groups				χ^2^	*P*
	Group R (n = 30)	Group D (n = 30)	Group E (n = 30)	Group S (n = 30)		
Dizziness	5(16.7)	6(20)	5(16.7)	7(23.3)	0.59	0.89
Nausea or vomiting	5(16.7)	6(20)	4(13.3)	7(23.3)	1.11	0.77
hypotension	2(6.7)	2(6.7)	1(3.3)	3(10)	1.07	0.78
Bradycardia	3(10)	4(13.3)	2(6.7)	3(10)	0.74	0.86

Group R: ropivacaine, Group D: ropivacaine with dexmedetomidine, Group E: ropivacaine with dexamethasone, Group S: ropivacaine with sufentanil. Data were presented as case number (%).

As detailed in Table 4, the intragroup comparison demonstrated the QoR-15 scores after surgery on POD1 were significantly higher in Group D (adjusted *P* value <0.0001), Group E (adjusted *P* value <0.0001), and Group S (adjusted *P* value = 0.0166) than in Group R. On POD2, there were significant differences between Group D (adjusted *P* value <0.0001), Group E (adjusted *P* value <0.0002) and Group R. Moreover, there were also significant differences between Group D and Group S at POD1(adjusted *P* value *P* = 0.0029) and POD2 (adjusted *P* value *P* < 0.0008). However, no significant differences were found between the groups in the QoR-15 scores on POD7 (*P* > 0.05).

**Table 4:** QoR-15 scores after surgery.

Postoperative day	Groups				*H*	*P*
	Group R (n = 30)	Group D (n = 30)	Group E (n = 30)	Group S (n = 30)		
**1**	105(100-119)	115(108–122) ^a^	113.5(106–118)^ a^	111(106–119) ^ab^	46.63	＜0.0001
**2**	114.5(104-124)	121(115–127) ^a^	120(115–125) ^a^	118(112–125) ^b^	41.67	＜0.0001
**3**	124.5(114-133)	127.5(121–136)^ a^	125(117-131)	125(118-134)	10.62	0.01
**7**	131(127-133)	132(126-138)	130.5(128-137)	131(127-136)	5.41	0.14

Group R: ropivacaine, Group D: ropivacaine with dexmedetomidine, Group E: ropivacaine with dexamethasone, Group S: ropivacaine with sufentanil. Data were presented as median (interquartile range). VAS, visual analogue scale.

a refers to intergroup comparisons against Group R (adjusted *P* value <0.05).

b refers to intergroup comparisons against Group D (adjusted *P* value <0.05).

## Discussion

Our trial demonstrated that the incorporation of adjunctive medications significantly prolonged the postoperative analgesic effects of the TPVB. Specifically, the combination of dexmedetomidine with ropivacaine reduced patient-reported pain scores at rest by 1 point and scores during coughing by 0.8 point at 12–20 h postoperatively. These reductions exceed the suggested value for minimal clinically important differences in pain scores [23]. Therefore, the addition of dexmedetomidine to ropivacaine may extend the duration of TPVB by ‌approximately 50–70%‌ in specific clinical settings, though this effect could vary depending on dosage, patient characteristics, or study design‌. In a previous study by Gao et al. [24], the authors found that by adding dexmedetomidine (1 µg/kg) to 0.5% ropivacaine, the block time of erector spinae plane block could be prolonged by approximately 120%. Marhofer also demonstrated that adding dexmedetomidine to 0.75% ropivacaine increased the duration of peripheral nerve block by 60% compared to ropivacaine alone [25]. Our findings are similar to those of previous studies. While bradycardia and hypotension are reported as among the more frequent adverse reactions associated with dexmedetomidine use‌ [26], no clinically significant occurrences of these events were observed in our study. This outcome may be influenced by the relatively lower dosage of dexmedetomidine administered, which aligns with evidence suggesting dose-dependent effects on hemodynamic stability‌. Dexmedetomidine has not been shown to exert neurotoxic effects when used for peripheral nerve blocks. However, the cardiovascular effects of higher doses remain a potential concern in patients with preexisting heart disease [27].

Dexamethasone is one of the most widely used adjuvants in local anaesthesia [16]. It has anti-inflammatory, analgesic, immunosuppressive, and antiemetic effects [28]. Our study found that the combination of dexamethasone (0.1 mg/kg) with ropivacaine could reduce pain scores at rest and during coughing within 12–16 h after surgery. Compared to ropivacaine alone, the addition of dexamethasone may extend the duration of TPVB by ‌approximately 20–30%‌ in specific clinical contexts‌. In another study, the addition of 8 mg of dexamethasone to local anaesthetics for brachial plexus block doubled the time to the first need for opioids [28]. Additionally, a recent RCT showed that dexamethasone with peripheral nerve block increased the analgesic duration by 3 h compared to intravenous dexamethasone, which is similar to our findings [29]. Dexamethasone, as an adjuvant for peripheral nerve blocks, was well tolerated by most patients. Some degree of neuroprotection and antihyperalgesia was also observed with clinically relevant doses of dexamethasone in animal models [16].

Preliminary evidence from our study indicates that combining sufentanil with ropivacaine in TPVB may be associated with a ‌prolongation of postoperative analgesia duration by approximately 20–30%‌ compared to ropivacaine monotherapy. This observed effect could be influenced by factors such as sufentanil dosage or patient-specific responses to opioid adjuvants. One reason for the enhanced analgesic effects may be the systemic absorption of the drug into the blood [22], while another reason may be the peripheral mechanism of action. However, a previous study showed that after paravertebral bolus and infusion administration, the maximum plasma drug concentrations of fentanyl and clonidine were lower than the effective levels after IV administration, suggesting that their analgesic effects may be partly attributed to a peripheral mechanism of action [22]. There is no uniform standard for the use of sufentanil, but some studies suggest that the concentration of 0.68 ug/ml may produce better analgesic effects, although it may also increase the incidence of adverse reactions such as nausea and vomiting [22]. In our study, a concentration of 0.5 μg/mL sufentanil was selected to assess its potential to enhance analgesic efficacy while mitigating adverse reactions‌. The findings suggested improved pain relief compared to baseline; and the observed numerical increase in nausea and vomiting incidence did not reach statistical significance when compared to ropivacaine monotherapy. (23.3% vs. 16.7%).

The co-administration of adjuvants with ropivacaine demonstrated significant clinical benefits in patients undergoing VATS, including a decreased consumption of postoperative opioids, later and less frequent PCIA use, and a faster time until ambulation. More importantly, our analysis of intergroup pairwise comparisons showed that dexmedetomidine was more effective than dexamethasone and sufentanil in reducing additional postoperative analgesics, the length of chest tube placement and overall length of hospital stay. As shown in a previous study, dexamethasone and dexmedetomidine both showed an overall nerve block duration up to 40% longer than that of the control. However, the requirement for postoperative analgesia was lower in the dexmedetomidine group than in the control and dexamethasone groups [14], which was consistent with the findings of the present study. This differential performance may relate to dexmedetomidine’s dual α2-adrenergic agonism and anti-inflammatory properties‌, contrasting with the single-action mechanisms of glucocorticoid receptor modulation (dexamethasone) and μ-opioid receptor activation (sufentanil)‌.

Additionally, we chose the QoR-15 score as the secondary outcome, which was the first measurement instrument of postoperative quality of recovery to undergo a systematic review according to the COSMIN checklist [30,31]. The observational data suggested a trend toward improved postoperative recovery in intervention groups, with total QoR-15 scores marginally exceeding control group values on POD1/POD2. Notably, the dexmedetomidine co-administered cohort exhibited a numerical elevation in global QoR-15 scores at POD1, POD2 and POD3 relative to dexamethasone and sufentanil comparators‌. Therefore, the current results tentatively support the hypothesis that dexmedetomidine with ropivacaine for TPVB may reflect potential advantages in early postoperative rehabilitation‌. This is consistent with previous research findings that perineural application of dexmedetomidine provides better analgesia and improves patient satisfaction [32,33].

Our results should be interpreted with caution because of several limitations. First, further large-scale trials are required to evaluate the clinical efficacy of local anaesthetic adjuvants. In addition, most data in our study were obtained via telephone after surgery. Although preoperative education was provided to the patients, some did not strictly comply with the study requirements, which may have affected the validity of the results. Finally, we conducted a 2-day postoperative pain follow-up for the participants based on several studies, which showed that postoperative pain peaked during this period [34]; however, this follow-up was too short to assess all relevant aspects of recovery.

## Conclusion

These findings suggest that adjuvant analgesia for TPVB can improve postoperative pain without obvious adverse reactions in patients undergoing VATS. However, compared with dexamethasone and sufentanil, dexmedetomidine is more effective in extending the duration of analgesia, reducing the demand of postoperative analgesics, improving the quality of recovery at 48 h postoperatively and shorting the length of hospital stay.

## Supporting information

S1 Checklist CONSORT checklist. (DOCX)

S1 File Study protocol. (DOCX)

S2 File Data used in analysis. (XLSX)

S3 File Assumptions of parametric tests. (PDF)
