# Supplementary material for: Comparison of different adjuvant analgesia for paravertebral block in video-assisted thoracoscopic surgery: A double-blind randomized controlled trial
Source: PLoS One. 2025 May 2;20(5):e0322589. doi: 10.1371/journal.pone.0322589 (PMC12047843; doi:10.1371/journal.pone.0322589)
Supplement: S1 File — (DOCX) [file pone.0322589.s002.docx]

**Project name:** **Comparison of different adjuvant analgesia for paravertebral block in video-assisted thoracoscopic surgery**

**The purpose and significance of the project**

**Research objectives:**

In recent years, ultrasound guided Thoracic paravertebral block (TPVB) has achieved significant advantages in postoperative analgesia after thoracic surgery, with high safety, low adverse reactions and complications. However, the analgesic time of ropivacaine nerve block is limited. Therefore, many clinical studies have been carried out on various analgesic strategies such as drugs, routes of administration, and interactions between drugs. Among them, the use of local anesthetic adjuvant is increasing, the main purpose is to enhance the analgesic effect and prolong the duration of analgesia.

Dexamethasone, dexmedetomidine and opioids are the most commonly used adjuvants in clinical nerve blocks, but their efficacy and safety have been controversial, and few studies have systematically compared the effects of the three adjuvants. In this study, dexamethasone, dexmedetomidine and sufentanil were used as adjuvants for ropivacaine to observe their effects in thoracoscopic surgery, and evaluate their effects on early postoperative pain and chronic postsurgical pain (CPSP), as well as the quality of early recovery within one month after surgery.

**Significance of the study:**

The analgesic effect of TPVB is considered to be the closest approach to thoracic epidural block. General anesthesia combined with TPVB will become the more recommended type of anesthesia for thoracic surgery. For patients who need to recover cough and sputum as soon as possible after surgery, TPVB guided by ultrasound is an ideal analgesic measure, which is accurate in location, safe in operation, and has less impact on hemodynamics, and significantly reduces the incidence of complications such as nerve damage and urine retention. As a new technology recommended by clinical anesthesiologists in recent years, TPVB is an ideal analgesic measure. Compared with traditional techniques, TPVB has unique advantages in perioperative analgesia in thoracic surgery, heart surgery, breast surgery, abdominal surgery, urology surgery and special groups.

Adjuvant drugs of local anesthetics are a relatively new method recently. There are many kinds of adjuvant drugs tried in clinical practice, but there is a lack of systematic comparison and evaluation of the effects of various drugs after use, especially prospective randomized controlled studies. This clinical study is strictly in accordance with the methods of randomized controlled trials. To evaluate and summarize the anesthetic and analgesic effects of various types of local anesthetic adjuvant combined with ropivacaine in TPVB in thoracoscopic surgery and its application prospects, in order to find a better ratio of nerve blocking drugs and postoperative analgesia methods.

So far, opioids occupy an important leading position in the field of pain drug treatment, and their clinical application is very wide. However, its side effects also bring a certain burden for the development of clinical work and the rapid recovery of patients after surgery, especially postoperative nausea and vomiting, lethargy, urinary retention, gastrointestinal peristalsis and so on. Therefore, in recent years, weak opioid or deopioid anesthesia has been advocated, emphasizing the use of local infiltration anesthesia, non-steroidal anti-inflammatory drugs, nerve block and other methods for intraoperative and postoperative analgesia. In this study, different adjuvant drugs combined with ropivacaine were used to perform nerve block technology to minimize perioperative opioid use and its side effects. At the same time, it is also expected to find the most reasonable and ideal drug compatibility for clinical anesthesiologists, and strive to achieve deopioid anesthesia.

The rapid rise of medical expenses has caused widespread concern in the society. How to choose the low-cost technology will become an unavoidable problem for health administration departments, hospitals and medical personnel. Under the advocacy of ERAS concept, this study hopes that through nerve block technology, the intraoperative and postoperative opioid consumption and side effects of patients can be reduced, the early movement of patients out of bed and functional recovery can be promoted, the workload of surgeons and nursing teams can be reduced, the length of hospital stay can be shortened, medical costs can be reduced, patients can benefit, and limited health resources can be utilized more rationally.

**Project Research Scheme**

**1. Design**

After the study was reviewed by the Ethics Committee of the Second Hospital of Lanzhou University, written informed consent was signed with the patients and their families. A randomized, blind design trial was conducted, with ropivacaine alone receiving TPVB as the control group, dexmedetomidine, dexamethasone and sufentanil combined with Ropivacaine receiving TPVB as the experimental group, respectively, to explore the effects of three drugs as local anesthetic adjuvant on the analgesic effect after TPVB block.

**2. Subjects and groups**

A total of 120 patients were selected for thoracoscopic surgery.

Inclusion criteria: (1) Gender was not limited, age was 18-70 years old; (2) BMI 18-28kg /m^2^; (3) ASA grades Ⅰ ~ Ⅲ; (4) Proposed surgery: thoracoscopic surgery.

Exclusion criteria: (1) known allergy to the test drug; (2) coagulation dysfunction; (3) infection of the puncture site; (4) Recent history of painkiller intake and drug abuse; (5) suffering from mental disorders and other diseases;

Exclusion criteria: serious complications or accidents of surgery or anesthesia in the perioperative period; Did not cooperate well with postoperative data collectors.

Experimental grouping: The patients were divided into four groups by random number table method: ropivacaine block group (group C), ropivacaine combined with dexmedetomidine group (group D), ropivacaine combined with dexamethasone group (group E), ropivacaine combined with sufentanil group (group S), with 30 cases in each group.

Method of grouping: Excel software was used by doctors who were not involved in nerve block operation and effect evaluation to generate and record all random numbers without duplication between 1 and 120. 120 random numbers represented the included 120 patients, in which multiple of 4 (4n) was group C patients, 4n+1 was group D patients, 4n+2 was group E patients, and 4n+3 was group S patients. After the grouping was completed, the group corresponding to each number and the intervention measures were sealed back plexus 1 and numbered to ensure that it corresponded to the order of the included patients one by one. On the day of the experiment, it was handed over to the doctor who performed nerve block operation for drug allocation and TPVB operation. Evaluation after the procedure was performed by physicians who were unaware of any of these procedures, and patients and family members were unaware of the grouping.

Primary instruments:

Portable 2D ultrasound instrument (sonosite M-Turbo®, sonosite, USA), ultrasound probe (5-15MHZ high frequency linear array probe), ultrasound coupler, sterile probe Kit, multifunctional anesthesia monitor (PHLIPS MP40, PHLIPS Inc., The Netherlands), Puncture needle (20G 100mm, Medizinth chnologle, Geisingen, Germany), 20ml syringe.

Drug configuration:

0.375% ropivacaine: (0.75% Ropivacaine 10ml+0.9% sodium chloride injection 10ml)

Dexmedetomidine: (0.375% ropivacaine mixed with 1ug/kg)

Dexamethasone: (0.375% ropivacaine compatible dose 0.1mg/kg)

Sufentanil: (0.5ug/ml in 0.375% ropivacaine)

Controlled intravenous analgesia (PCIA) : (hydromorphone 0.1mg/ml+ Butorphinol tartrate 50ug/ml+ Ondansetron 16mg+0.9% normal saline diluted to 100m1, background dose was 2.5ml/h, self-controlled supplemental dose was 2m1, locking time was 15min)

**3. Method**

3.1 Preparation before anesthesia

Venous access was opened after the patient entered the room, and SBP, DBP, MAP, HR, ECG, ECG were routinely monitored.

SpO2, radial artery puncture catheterization under local anesthesia. The puncture site can be selected on the more convenient side according to the surgical position during the operation, but Allan test should be performed before insertion. If the result is positive, the puncture on this side should not be performed and the site should be changed. After the puncture was completed, a blood gas analysis was performed first, which was used as the reference value of the subsequent blood gas analysis results, and TPVB was induced before anesthesia.

3.2 Intervention Measures

Both groups had TPVB on the right side under ultrasound guidance. Specific operations were as follows:

The patient is placed in a prone position and the probe is placed in a sagittal position at T5 level, approximately 2.5cm from the midline, using a portable ultrasound instrument. The midpoint of the probe is located between two adjacent transverse processes, thus placing the target thoracic paravertebral space in the center of the ultrasound image. After locating the pleura and superior costal transversal ligaments, the puncture needle is inserted into the head from the lower edge of the probe along the sagittal direction, keeping the needle body always in the plane of the ultrasound image, and the direction of injection can be adjusted appropriately under the guidance of ultrasound. When the needle tip enters the paravertebral space through the superior costal transversal ligaments, there may be a sense of loss of resistance. If there is no blood or air after extraction, Then the experimental drug could be slowly injected into each group (0.375% ropivacaine 20ml in group C; Group D was given dexmedetomidine 1ug/kg+0.375% ropivacaine 20ml; Group E was dexamethasone 0.1mg/kg+0.375% ropivacaine 20ml; In group S, Sufentanil 0.5ug/ml+0.375% ropivacaine 20ml. After injection, the diffusion of local anesthetics can be seen on ultrasound images, and the subpleural pressure sign will be more clear about the block site. TPVB was performed by the same skilled anesthesiologist in all patients. The sensory block level was measured by alcohol swabbing by an anesthesiologist who was unaware of the test drug at 5-minute intervals until 30 minutes after injection. Anesthesia induction was initiated when no abnormal reactions such as local anesthetic intoxication were observed.

3.3 Intraoperative and postoperative management

Anesthesia was induced intravenously, with midazolam 0.05mg/kg, propofol 1.5-2.0mg/kg, Sufentanil 0.5ug/kg, and cisatracurium 0.2mg/kg in sequence. Mask assisted breathing for 3min. After the patient's consciousness disappeared and the jaw was completely relaxed, a double-lumen bronchial catheter was inserted, and mechanical ventilation was performed after adjustment to the optimal position with the assistance of bronchoscopy. Single lung ventilation was performed 5min before the operation began, volumetric controlled ventilation mode and lung protective ventilation strategy were adopted during the operation, and tidal volume was set at 6-8 mL /kg. Respiratory rate was 14-20 times /min, PEEP value was set at 3-5cmH2O, inhaled oxygen concentration was 50%-70%, and inhaled oxygen flow was 1-2L /min. During the operation, respiratory parameters could be adjusted to maintain PaCO2 at 37-40mmHg and SpO2 > 90%, so as to avoid hyperventilation and hyperco2-emia.

Anesthesia maintenance: intravenous injection of remifentanil 0.15-0.3µg/kg/min and propofol 4-6 mg/kg/h, BIS value between 40 and 60, intermittent infusion of cis-atracurium to maintain muscle relaxation. Vasomotor drugs (norepinephrine, atropine, ephedrine) were prepared during the operation, and the dosage of each drug was reasonably adjusted according to the operation conditions, so that the fluctuation range of HR and MAP did not exceed ±20% of the basic value. After the operation, manual pulmonary revascularization was performed, and ondanseetron 8mg was given intravenously and PCIA was activated before the double-lumen tube was removed.

Patients were sent to PACU for observation for at least 1 hour after surgery, and VAS scores were performed at each time point. If the patient's VAS score was greater than 4 and additional analgesia was needed, 0.2 mg of hydromorphone and 100µg of butorphorphine were injected intravenously via PCIA to relieve pain, and the patient was locked up for 15 minutes. If the pain does not relieve after PCIA, 60mg ketochromate ambutrin can be administered intravenously, which should be halved to 30mg for patients over 65 years of age or weighing less than 50kg. In severe nausea and vomiting, ondansetron 4 to 8mg intravenously is given. All patients were required to complete the 15-item Quality of Recovery Score Scale (QoR-15) and APS-POQ-R questionnaire within one month after surgery.

3.4 Management of adverse events

In the course of nerve block, if local anesthetic intoxicants occur, the administration should be stopped immediately, oxygen should be given by mask to maintain airway patence, midazolam (0.05 ~ 0.1mg/kg) or propofol (1 ~ 2mg/kg) can be injected intravenously. When circulation is inhibited, Blood volume should be supplemented as soon as possible or vasoactive drugs (norepinephrine, atropine, ephedrine) should be applied to maintain hemodynamic stability; If respiratory depression occurs, oxygen should be given by mask pressure immediately, and effective artificial ventilation should be carried out to maintain SpO2 and PaCO2 within the normal range. If bradycardia occurs (HR < 50 beats/min), atropine 0.05mg/kg can be given; For severe nausea and vomiting, ondansetron 8mg. If massive bleeding is caused by the operation during the operation, blood transfusion and infusion should be performed immediately, vasoactive drugs should be pumped to maintain circulation if necessary, blood gas analysis should be performed timely and repeatedly, and internal environment should be kept relatively stable as far as possible.

3.5 Data Collection

(1) Record the patient's gender, age, BMI, anesthesia time, operation time and other general information;

(2) The plane range of sensory block within 30 minutes after successful puncture, and the incidence of complications related to puncture, including local anesthetic intoxication, nausea, vomiting, respiratory depression, hypotension, bradycardia, etc.;

(3) Total intraoperative opioid use;

(4) VAS scores at 4h (T1), 8h(T2), 12h (T3), 16h (T4), 20h (T5), 24h (T6) and 48h(T7) after surgery. Visual analog Scale (VAS) score: Draw a 10cm horizontal line on the paper, one end of the horizontal line is 0, indicating no pain; The other end of the line is 10, indicating severe pain; The middle part indicates varying degrees of pain;

(6) the number of analgesic pump compressions and the total amount of ketone chromate troamine used within 48 hours after surgery and the occurrence of adverse reactions;

(7) The 15-item Recovery Quality Score Scale (QoR-15) and the results of APS-POQ-R questionnaire were recorded within one month after the operation.

3.6 Test suspension criteria

During the course of the test, the subject is required to discontinue the test if the following conditions occur: The reasons for discontinuation of the study should be recorded and evaluated in detail on the observation sheet.

(1) Patients and their families insist on withdrawing from the trial during the course of the trial;

(2) TPVB failed in the implementation process under the guidance of ultrasound, including multiple puncture or poor diffusion position of the liquid after injection, or local anesthetic poisoning and other adverse reactions;

(3) Adverse events such as massive bleeding or cardiac arrest occurred during the operation, or the surgical mode changed from thoracoscopic lobectomy to thoracotomy;

(4) Insufficient postoperative follow-up time or incomplete data collection, which affected the overall data collection and analysis

(5) Postoperative subjects could not follow the medication requirements specified in the trial setting protocol;

(6) If the patient has incision site infection, split, persistent high fever, etc., within 48 hours after surgery, or if another operation is required due to other factors, the current trial should be discontinued;

**2.** Existing risks and intervention measures

In the course of nerve block, if local anesthetic poisoning occurs, the administration should be stopped immediately, oxygen should be given through a mask to maintain airway patence, midazolam (0.05-0.1 mg/kg) or propofol (1-2mg /kg) can be injected intravenously. Blood volume should be supplemented as soon as possible or vasoactive drugs (norepinephrine, atropine, ephedrine) should be applied to maintain hemodynamic stability; If respiratory depression occurs, oxygen should be given by mask pressure immediately, and effective artificial ventilation should be carried out to maintain SpO2 and PaCO2 within the normal range. If bradycardia occurs (HR < 50 beats/min), atropine 0.05mg/kg can be given; For severe nausea and vomiting, ondansetron 8mg. If massive bleeding is caused by the operation during the operation, blood transfusion and infusion should be performed immediately, vasoactive drugs should be pumped to maintain circulation if necessary, blood gas analysis should be performed timely and repeatedly, and internal environment should be kept relatively stable as far as possible.

1. Statistical analysis

Statistical analyses were performed using SPSS software (version 25.0; IBM, USA). Assumptions of parametric tests (normality, homogeneity of variance) were verified using Shapiro-Wilk and Levene's test‌. The measurement data were expressed as mean ± standard deviation (SD) or median [inter-quartile range (IQR)]. The count data were expressed as frequencies or percentages. A multigroup comparison of the normally distributed measurement data with homogeneity of variance was conducted using one-way analysis of variance (ANOVA), and the Tukey-Kramer method was used to compare the intergroup pairwise. The Kruskal-Wallis H-test was employed to compare measurement data that did not adhere to a normal distribution or exhibited heterogeneous variance among multiple groups. And the Dunn's test was utilized for intergroup pairwise comparisons. The original p-value was adjusted using the Bonferroni method, thereby more rigorously controlling the type I error rate. The Chi-squared (c2) test was used to compare categorical and count data, and P < 0.05 was considered statistically significant.

**Qualification and introduction of drugs or equipment involved**

The main drugs used are ropivacaine, sufentanil, dexmedetomidine, dexamethasone and conventional narcotic drugs. The project leader and major participants have the qualification certificate of narcotic drug use training and corresponding prescription rights.

**Informed Consent Form for Patient Participation**

**Study name: Comparison of different adjuvant analgesia for paravertebral block in video-assisted thoracoscopic surgery**

**Protocol Version No.: Protocol Version Version 2.0 Date: January 1, 2022**

**Research Center: The Second Hospital of Lanzhou University**

**About:**

We cordially invite you to participate in this prospective study, which is a clinical study. You are invited to participate in this study because you are about to undergo radical surgery for Ivor-Lewis esophageal cancer. Before participating in the study, you may read and understand the contents of this patient informed consent form, which describes the purpose, process, possible benefits and risks of the study, and your rights as a participant. You need to read this informed consent carefully to be able to fully understand its contents. After reading this Informed Consent form, discuss it with your attending physician. If you decide to participate in the study, you will need to sign this informed Consent form and you will be provided with a signed copy of the informed Consent form.

**Purpose of the study:**

The purpose of this study was to collect relevant case data and experimental data for systematic analysis, to evaluate the blocking effect of different types of adjuvants combined with ropivacaine in TPVB and the influence on postoperative pain and recovery quality; To determine which type of adjuvant can produce the most clear effect, achieve the ideal analgesic effect with fewer complications, and seek new methods and ideas for anesthesia and analgesia for patients undergoing radical surgery for esophageal cancer.

The study involved 120 patients.

Conditions for participation in this study:

1. Signing informed consent;
2. Any gender, age 18-70 years old;
3. BMI 18-28kg /m^2^;
4. ASA grades Ⅰ ~ Ⅲ;
5. Intended surgery: thoracoscopic surgery.

The following conditions preclude participation in this study:

1. A known allergy to the trial drug;
2. Clotting dysfunction;
3. Puncture site infection;
4. Recent history of painkiller intake and drug abuse;
5. Suffering from mental disorders or other medical conditions;

**Do I have to participate in this study?**

No. Participation in this study is entirely voluntary. You may agree or decline to participate in the study, or withdraw at any time during the study. Your decision does not affect your future treatment.

**A: What do I need to do?**

First visit after you agree and sign this Informed Consent form. At a later time (planned for a month or more), you will need to complete a number of recovery quality questionnaires (including pain levels, sleep conditions, mental state, etc.) and briefly ask a few questions about your post-op situation. At present, nerve block has been found to be effective in relieving postoperative pain. We hope to further study these methods and techniques in order to better reduce postoperative pain. To guide the future pain management and treatment of this kind of surgery.

The specific follow-up time of this study is determined according to your actual treatment needs. The following information will be collected: age, gender, height, weight, intraoperative vital signs and postoperative pain score. If you do not agree to provide the above information, you will not be included in the study.

There will be no intervention in the diagnosis, treatment, examination or visit of the disease, and you will not receive additional tests or treatment as a result of participating in the study.

**Are there any potential risks associated with participating in this study:**

Any treatment comes with its own risks, and your attending doctor will talk to you about the possible risks of treatment. This study does not interfere with your doctor's choice of treatment, only information about the disease and treatment is collected, and there are no risks outside of normal treatment associated with participating in this study.

**Benefits of participating in this study:**

Participating in this study can facilitate your regular visit to the doctor and help you to grasp the changes of your condition in time, which is crucial for disease control and benign outcome. At the same time, your participation may be helpful to the further investigation of the nature of the disease, which will certainly greatly promote the development of medicine. Ultimately, it will allow you to make progress in the treatment and rehabilitation of the disease, while also benefiting others in your family who may have inherited the disease.

**Am I paid to participate in this study?**

You are not paid to participate in the study. However, you will not have to bear any additional costs for participating in this study.

**Will my information be kept confidential?**

Your personal information will be kept confidential and kept in a database that is not open to the public. Reviewers of the relevant government regulatory agencies or personnel of the ethics committee may be authorized to view your data to ensure the integrity of the research process and data accuracy.

Your data will be integrated with the data of other patients participating in the study for scientific purposes, and by signing this document, you are giving us (or other staff involved in the study) permission to use your data for research purposes. The authorization for this study does not have an end date.

**Who to contact in the future?**

If you have any questions about this study, or experience discomfort during treatment, contact the attending physician.

The trial was reviewed and approved by an ethics committee.

Ethics Committee contact: 0931-8942234

**Informed Consent and Authorization Statement**

**Study name: Comparison of different adjuvant analgesia for paravertebral block in video-assisted thoracoscopic surgery: A Double-Blind Randomized Controlled Trial**I have read and understood the informed consent form, which clearly explains the issues related to this study and my involvement. By signing this statement, I voluntarily consent to participate in the scientific study, and I consent to the investigator's use of my diagnosis and treatment data in accordance with this informed consent for the study and use of this data for scientific purposes.

This is to be completed only by the patient or the patient's representative.

Block name

Signing date

Relationship with the patient (agent)

Block name

Signing date

Confirmation statement from the informed consent person

I have explained this study to the above patients. The patient has been fully informed of this information and has consented to participate in the study.

Research Doctors

Block names

Signing date
