# Supplementary material for: Comparison of different adjuvant analgesia for paravertebral block in video-assisted thoracoscopic surgery: A double-blind randomized controlled trial
Source: PLoS One. 2025 May 2;20(5):e0322589. doi: 10.1371/journal.pone.0322589 (PMC12047843; doi:10.1371/journal.pone.0322589)

Age (ANOVA)

Shapiro-Wilk test:

W 0.9699 0.9458 0.9481 0.9714

P value 0.5372 0.1306 0.1501 0.5780

Levene's tests:

F* (DFn, DFd) 1.104 (3.000, 110.2)

P value 0.3506

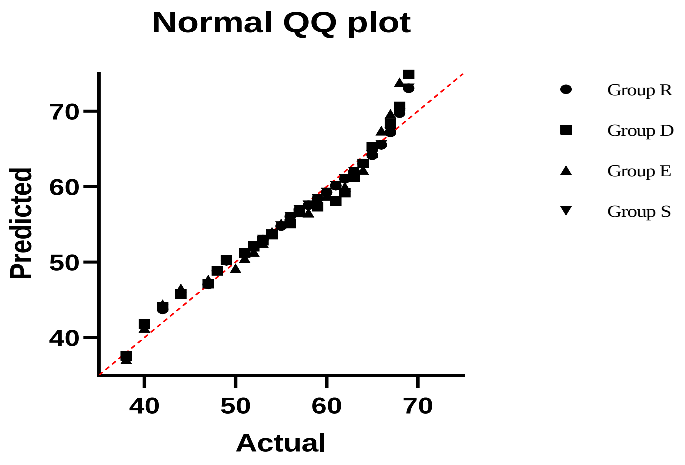

Weight(ANOVA)

Shapiro-Wilk test:

W 0.9502 0.9681 0.9462 0.9528

P value 0.1713 0.4873 0.1334 0.2009

Levene's tests:

F* (DFn, DFd) 0.05207 (3.000, 115.7)

P value 0.9842

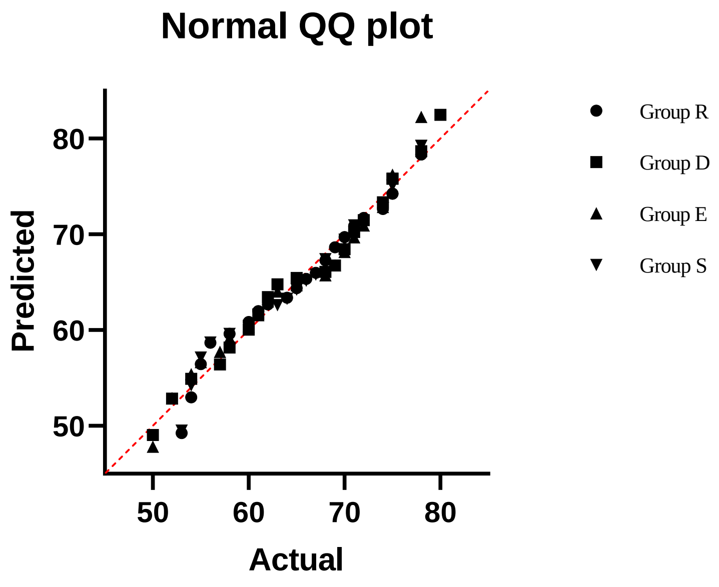

Height: (ANOVA)

Shapiro-Wilk test

W 0.9412 0.9361 0.9429 0.9418

P value 0.0980 0.0714 0.1088 0.1015

Levene's tests:

F* (DFn, DFd) 0.1540 (3.000, 112.4)

P value 0.9269

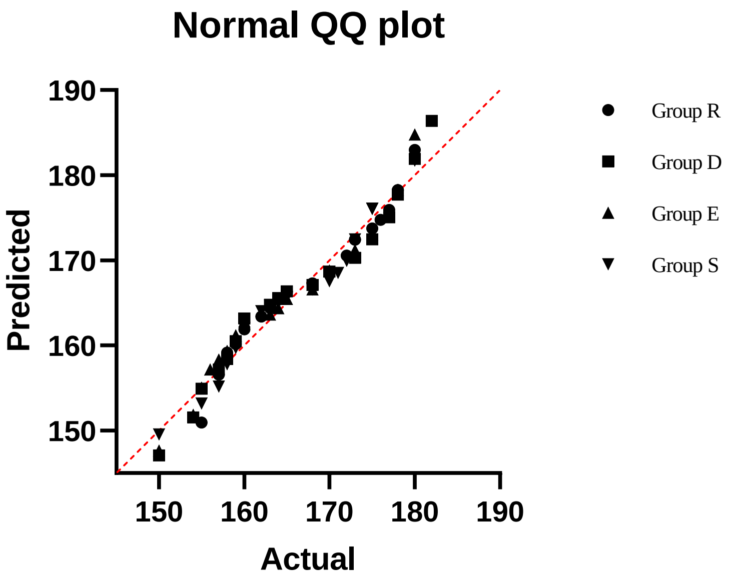

Anesthesia Duration (ANOVA)

Shapiro-Wilk test

W 0.9644 0.9612 0.9543 0.9490

P value 0.3986 0.3327 0.2200 0.1588

Levene's tests:

F* (DFn, DFd) 0.3191 (3.000, 113.2)

P value 0.8116

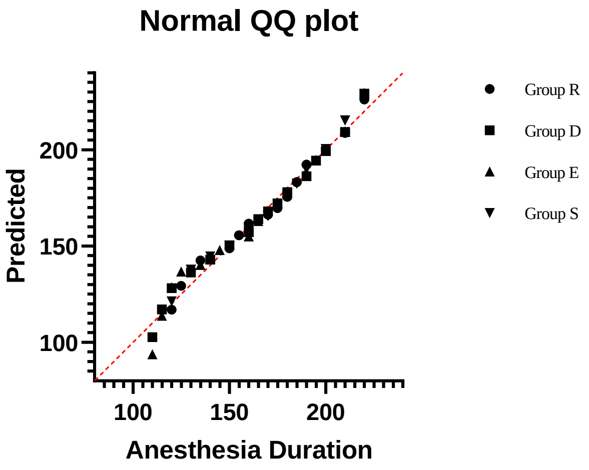

Surgery Duration (ANOVA)

Shapiro-Wilk test

W 0.9662 0.9637 0.9801 0.9596

P value 0.4416 0.3838 0.8270 0.3029

Levene's tests:

F* (DFn, DFd) 0.2660 (3.000, 111.5)

P value 0.8498

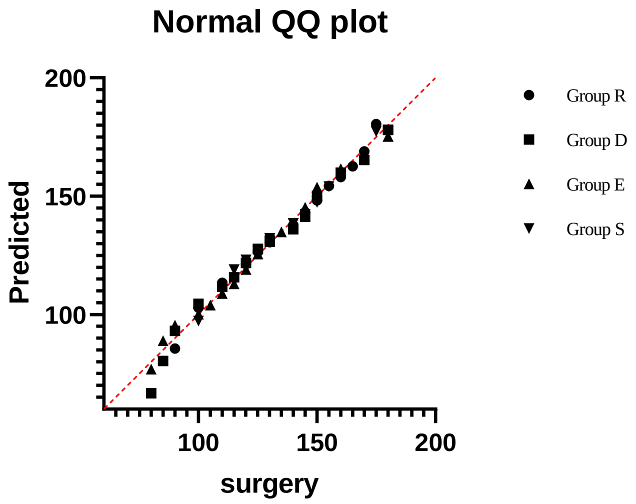

VAS scores (4h at rest): Kruskal-Wallis H-test

Shapiro-Wilk test

W 0.7403 0.7851 0.7034 0.6190

P value <0.0001 <0.0001 <0.0001 <0.0001

Levene's tests:

F* (DFn, DFd) 0.2168 (3.000, 110.1)

P value 0.8846

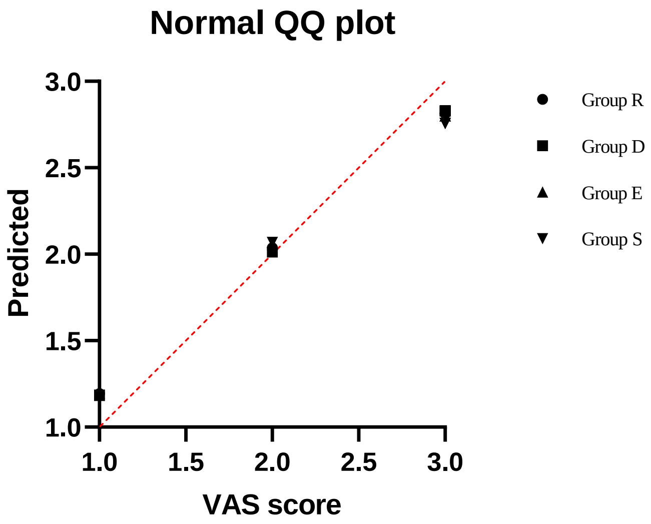

VAS scores (4h at coughing): Kruskal-Wallis H-test

Shapiro-Wilk test

W 0.6318 0.7512 0.6236 0.6382

P value <0.0001 <0.0001 <0.0001 <0.0001

Levene's tests:

F* (DFn, DFd) 1.463 (3.000, 113.2)

P value 0.2283

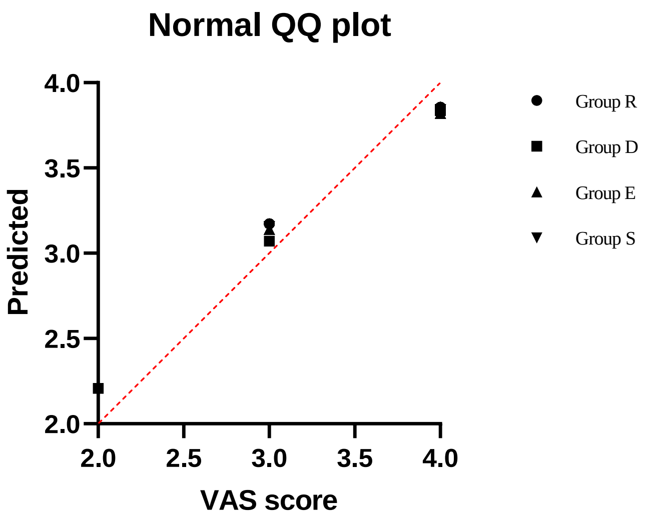

Chest tube: Kruskal-Wallis H-test

Shapiro-Wilk test

W 0.8586 0.8895 0.9149 0.9159

P value 0.0009 0.0047 0.0199 0.0211

Levene's tests:

F* (DFn, DFd) 3.668 (3.000, 112.8)

P value 0.0144

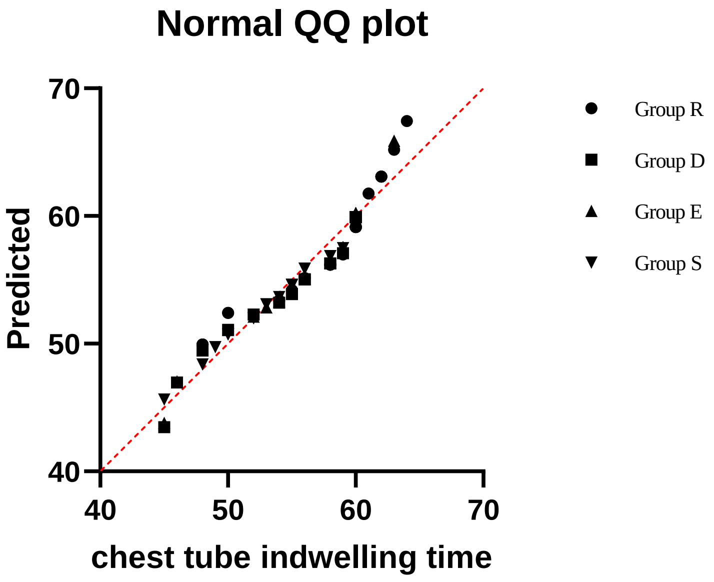

QoR-15 Score: Kruskal-Wallis H-test

Shapiro-Wilk test

W 0.9381 0.9702 0.9674 0.9222

P value 0.0809 0.5435 0.4697 0.0306

Levene's tests:

F* (DFn, DFd) 25.93 (3.000, 102.0)

P value <0.0001

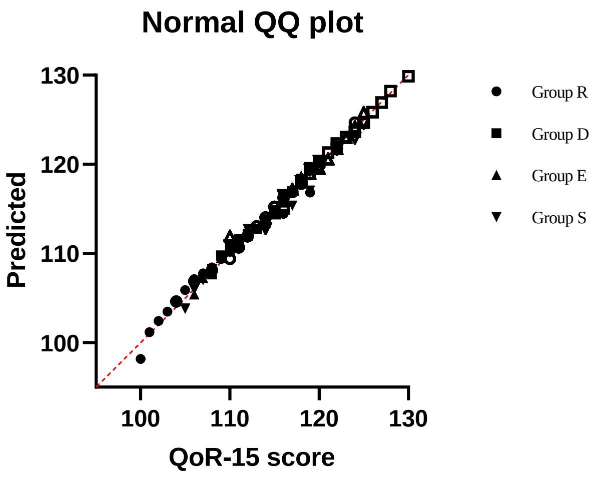

Length of hospital stay

Shapiro-Wilk test

W 0.9068 0.8795 0.8676 0.9080

P value 0.0123 0.0027 0.0015 0.0132

Levene's tests:

F* (DFn, DFd) 3.422 (3.000, 112.5)

P value0.0197

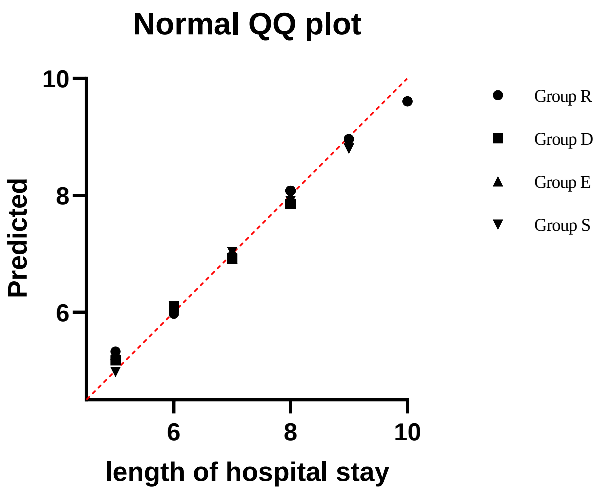

Supplement: S3 File — (PDF) [file pone.0322589.s004.pdf]
